# Disruption of microRNA Biogenesis Confers Resistance to ER Stress-Induced Cell Death Upstream of the Mitochondrion

**DOI:** 10.1371/journal.pone.0073870

**Published:** 2013-08-19

**Authors:** Karen Cawley, Susan E. Logue, Adrienne M. Gorman, Qingping Zeng, John Patterson, Sanjeev Gupta, Afshin Samali

**Affiliations:** 1 Apoptosis Research Centre, National University of Ireland, Galway, Ireland; 2 School of Natural Sciences National University of Ireland, Galway, Ireland; 3 MannKind Corporation, Valencia, California, United States of America; 4 School of Medicine, Clinical Science Institute, National University of Ireland, Galway, Ireland; University Health Network, Canada

## Abstract

Global downregulation of microRNAs (miRNAs) is a common feature of human tumors and has been shown to enhance cancer progression. Several components of the miRNA biogenesis machinery (XPO5, DICER and TRBP) have been shown to act as haploinsufficient tumor suppressors. How the deregulation of miRNA biogenesis promotes tumor development is not clearly understood. Here we show that loss of miRNA biogenesis increased resistance to endoplasmic reticulum (ER) stress-induced cell death. We observed that HCT116 cells with a DICER hypomorphic mutation (Exn5/Exn5) or where DICER or DROSHA were knocked down were resistant to ER stress-induced cell death. Extensive analysis revealed little difference in the unfolded protein response (UPR) of WT compared to Exn5/Exn5 HCT116 cells upon ER stress treatment. However, analysis of the intrinsic apoptotic pathway showed that resistance occurred upstream of the mitochondria. In particular, BAX activation and dissipation of mitochondrial membrane potential was attenuated, and there was altered expression of BCL-2 family proteins. These observations demonstrate a key role for miRNAs as critical modulators of the ER stress response. In our model, downregulation of miRNA biogenesis delays ER stress-induced apoptosis. This suggests that disrupted miRNA biogenesis may contribute to cancer progression by inhibiting ER stress-induced cell death.

## Introduction

Endoplasmic reticulum (ER) stress results from an accumulation of misfolded proteins in the ER lumen, which then evokes the unfolded protein response (UPR) [1]. Three ER transmembrane proteins, PRK (RNA-dependent protein kinase) like ER kinase (PERK), Activating Transcription Factor 6 (ATF6), and Inositol Requiring enzyme 1α (IRE1α) monitor the “health” of the ER [2]. Upon accumulation of unfolded proteins in the ER lumen, GRP78 dissociates from PERK, ATF6 and IRE1 permitting their activation and triggering UPR signals. Recent research has shown increasing complexity to the activation of these receptors and requires further events after GRP78 dissociation where unfolded proteins bind to this MHC-like grove in IRE1, promoting the formation of higher order oligomers required for UPR activation. The luminal domain of PERK and IRE1 shows similar features and thus are believed to be activated in a similar manner [3]. Initially UPR signals are prosurvival aiming to clear the ER of unfolded proteins and restore normal functioning. However, if stress is too severe adaptive signaling is insufficient and switches to promote cell death [4].

MicroRNAs (miRNAs) are small non-coding RNAs that function as endogenous effectors of RNA interference. As such they regulate protein expression via blocking mRNA to protein translation [5]. Two key enzymes needed for maturation of these small regulators are the RNase III enzymes DROSHA and DICER which cleave miRNAs at the base of the miRNA stem and finally removing the hairpin loop respectively thus forming the mature miRNA duplex of 21 nt [6].

ER stress-induced cell death relies upon intrinsic mitochondrial-mediated death signals to trigger cellular demise. Regulation of pro-survival and pro-apoptotic BH3-only proteins of the BCL-2 family leads to activation of mitochondrial-mediated death signals are key events in ER stress-induced apoptosis [7,8]. To date, most studies examining these death signals have focused primarily on transcription factor-dependent pathways with CHOP, a transcription factor activated via the PERK arm of the UPR, thought to mediate pro-death signals [9–11]. However, CHOP null MEF cells retain sensitivity to ER stress-induced death, albeit at a delayed rate, indicating that other pro-apoptotic mechanisms are at play [12]. Recently, a potential role for miRNAs as regulators of UPR and ER stress-induced death signaling has emerged. Downregulation of the miR-199a/214 cluster has been reported to increase XBP1s levels in hepatocellular carcinoma (HCC) which was linked to enhanced proliferation and tumor formation [13]. In a similar manner, PERK-NRF2 mediated downregulation the miR-106b-25 cluster has been linked to the induction of the pro-apoptotic BH3-only protein BIM [14].

Emerging evidence indicates miRNAs have important roles in cancer, with differential expression or total loss of miRNAs reported in a variety of cancers including breast, bladder and colon, as well as some leukemias [15–18]. Studies have demonstrated that loss of miRNAs can benefit cancer cells by enhancing tumorigenesis and modulating cells responsiveness to therapies, as seen by the repression of miR-15a/16-1 to increase BCL-2 expression and survival of CLL cells [19] and downregulation of p27^kip1^ by miR-221-222 in tamoxifen-resistant breast cancer cells [20]. In addition to this, the expression of DICER, an RNase III enzyme required for the processing and maturation of miRNA [6], is decreased in many cancers [21–24] where it was associated with enhanced tumorigenesis and poor patient prognosis [25–27]. DICER expression can also be decreased by an assortment of cellular stresses routinely encountered by tumors such as reactive oxygen species, phorbol esters, Ras oncogene signaling, type 1 interferons and serum withdrawal [28,29]. More recently, the anti-cancer effects of Metformin have been linked to increased DICER expression [30].

The UPR stress response mechanism is frequently hijacked by cancer cells as a means by which to survive the stressful conditions associated with tumor formation [31,32]. Given the emerging role miRNAs in cancer progression [33,34] we wished to study whether miRNAs could function as mediators of ER stress signals and ascertain if targeting miRNAs could represent a novel therapeutic strategy. To address this we opted to determine the effect of globally suppressing miRNA biogenesis in cells rather than targeting specific miRNA candidates. Here we report that loss of DICER and thus mature miRNAs attenuates ER stress-induced cell death in HCT116 cells through a mechanism involving the regulation of BCL-2 family members.

## Materials and Methods

### Cell culture and treatments

HCT116 and RKO, WT and DICER Exn5/Exn5 cells were a kind gift from Prof Bert Vogelstein and Victor E. Velculescu [35]. All cells described in this section were maintained in 75 cm^2^ flasks in McCoys 5A modified medium with 10% heat inactivated fetal bovine serum (FBS) and Penicillin/Streptomycin.

HCT116 WT cells were virally transduced with a GFP lentiviral vector carrying DROSHA targeting shRNA (pTIG-U6tetO-shDR) a kind gift received from Dr John Rossi [36], and were cell sorted based on GFP positivity, on a FACS ARIA flow cytometer. HCT116 WT cells were virally transduced with pSicoR- empty vector (EV) -puromycin and a pSicoR- puromycin DICER targeting shRNA vector lentivirus (Addgene 14763_Tyler Jacks lab). These cells were then selected using 3 µg/ml of puromycin, and maintained in 500 ng/ml of puromycin.

Where indicated cells were treated with 500 ng/ml of brefeldin A (Bfa), 500 ng/ml of tunicamycin (Tm), 200 nM of staurosporine (Sts), or 100 µM of etoposide (Etop) for the indicated time periods. All reagents were from Sigma-Aldrich unless otherwise stated.

### RNA Isolation

Total RNA was isolated using TRI Reagent (Invitrogen) by following the manufacturer’s instructions. Briefly, cells were scraped, collected and centrifuged at 1,500 rpm for 5 min. The pellet was then resuspended in TRI reagent. To separate RNA from DNA and protein, chloroform was added, mixed well and incubated for 2 min at room temperature. Samples were centrifuged at 12,000 g for 15 min. The upper clear phase was carefully extracted with special care taken not to disturb the interface. Isopropanol was used to precipitate RNA, overnight at -20^o^C. Samples were then centrifuged at 12,000 g for 10 min and the RNA pellet was washed in 75% ethanol. Ethanol was carefully removed and the pellet air dried. The RNA was resuspended in 25 µl of DEPC water and stored at -80^o^C.

### Reverse transcription RT-PCR

To synthesise cDNA, 2 µg of RNA was subjected to DNase treatment followed by EDTA inactivation (Invitrogen) (to ensure there was no contaminating genomic DNA present), and the RNA was then reverse transcribed into cDNA using Superscript III first strand RT-PCR system and random hexamers (Invitrogen) by following the manufacturer’s instructions.

### miRNA specific Reverse Transcriptase-PCR

RNA was diluted to 100 ng/µl and reverse transcribed into cDNA using miRNA specific primers and a miRNA reverse transcription kit (Applied Biosystems). PCR conditions: 16^o^C for 30 min, 42^o^C for 30 min and 85^o^C for 5 min.

### Quantitative Reverse Transcriptase-PCR

For qRT-PCR, cDNA was synthesized and diluted to a concentration of 40 ng per 10 µl reaction; it was then combined with Brilliant III Ultra-Fast QPCR Master Mix (Agilent) and 20X Taqman gene expression assays and accurately dispensed to a fast optical MicroAmp 96-well plate in triplicate (all supplied by Applied Biosystems). PCRs were run for 40 cycles on an Applied Biosystems fast 7500 machine using the following cycling conditions: Hold at 95^o^C for 3 min, then 40 cycles at 95^o^C for 12 s, and 60^o^C for 30 s. The relative expression levels (relative to GAPDH) were calculated using the △△Ct method [37] when comparing a treated sample to an untreated sample.

### Annexin V/ Propidium Iodide staining

Externalization of phosphatidylserine (PS) to the outer leaflet of the plasma membrane of apoptotic cells was assessed with Annexin V-fluorescein isothiocyanate (FITC), which has been expressed, purified and conjugated in-house as described by 38. The cells were collected and resuspended in ice-cold calcium buffer (10 mM HEPES/NaOH, pH 7.4, 140 mM NaCl, 2.5 mM CaCl_2_), and incubated with Annexin V-FITC for 15 min on ice. Prior to analysis 300 µl of calcium buffer containing 4 µl of PI (50 µg/ml) was added and cells analyzed on a FACSCanto flow cytometer.

### Measurement of ΔΨ_m_ by TMRE staining

Mitochondrial transmembrane potential (ΔΨ_m_) was determined by using the fluorescent probe tetramethylrhodamine ethyl ester (TMRE, Molecular Probes) as previously described [39]. Briefly, cells were trypsinized and incubated with TMRE at room temperature for 30 min in the dark and analyzed by flow cytometry using a FACS Canto flow cytometer.

### Western Blotting

Cells were lysed in whole cell lysis buffer (20 mM HEPES pH 7.5, 350 mM NaCl, 0.5 mM EDTA, 1 mM MgCl_2_, 0.1 mM EGTA, and 1% NP-40), boiled at 95 °C with Laemmli’s SDS-PAGE sample buffer for 5 min. Protein samples were separated by SDS polyacrylamide gel electrophoresis, transferred onto nitrocellulose membrane and blocked with 5% milk in PBS containing 0.05% Tween. The membrane was incubated in a 1:1000 dilution of primary antibody in 5% milk in PBS containing 0.05% Tween, caspase-3 (Cell Signaling Technology, Cat# 9662), caspase-9 (Cell Signaling Technology, Cat# 9504), PARP (Cell Signaling Technology, Cat# 9542), XBP1s (Biolegend), CHOP (Santa Cruz Biotechnology, Inc, Cat# sc-793), phosphorylated eIF2α (Cell Signaling Technology, Cat# 3597), total eIF2α (Cell Signaling Technology, Cat# 2103), IRE1α (Cell Signaling Technology, Cat# 3294S), BIM (Stressgen Cat # AAP-330), BAD (Santa Cruz Cat # sc-7869), BID (Santa Cruz cat # sc-11423), BAK (Santa Cruz Cat # sc-832), BCL-2 (Santa Cruz Cat # sc-509), MCL-1 (Cell signaling technologies Cat # 4572), BCL-x_L_ (Santa Cruz Cat # sc-8392), or β-ACTIN (Sigma, Cat# A-5060) overnight at 4 °C. They were further incubated in a 1:5000 dilution of appropriate horseradish peroxidase-conjugated secondary antibody (Pierce) in 5% milk in PBS containing 0.05% Tween. for 90 min. Signals were detected using West Pico chemiluminescent substrate (Pierce).

### Detection of active BAX by flow cytometry

Cells were trypsinized and then fixed in 2% formaldehyde for 10 min at room temperature. Washed cells were resuspended in PBS and stored at 4^o^C overnight. Anti-BAX antibody (BD Biosciences clone 6A7, 1 µg) was added to 100 µl of permeabilisation buffer (0.1% saponin, 0.5% BSA in 1x PBS) in which, cells were incubated for 1 h at 4^o^C. Mouse IgG isotype control (Biolegend) served as an autofluorescence control. Samples were washed and incubated with a 1:200 dilution of FITC-conjugated anti-mouse antibodies in PBS for 1 h at 4^o^C. Samples were washed and resuspended in 300 µl PBS and analyzed by flow cytometry. Histograms were overlaid using Cyflogic.

### Statisical Analysis

Experiments were repeated independently at least 3 times. Error bars represent the standard error of mean (SEM) of replicates. Significance was determined using a two-tailed Student’s t test, with *P*-value of less than 0.05 being considered significant and annotated by *.

## Results

### Disruption of Dicer impairs biogenesis of miRNA

ER stress and the resulting UPR pathway are known to induce an intricate transcriptional program. Interestingly, we observed that upon ER stress-induced apoptosis, in HCT116 cells treated with 500 ng/ml of Tm and 500 ng/ml of Bfa for 24 h, the expression of genes associated with miRNA biogenesis (DICER, DROSHA, TARBP1, AGO-2, AGO-4) were increased (Cawley, Gupta, Samali et al., unpublished data).

**Figure 1:**
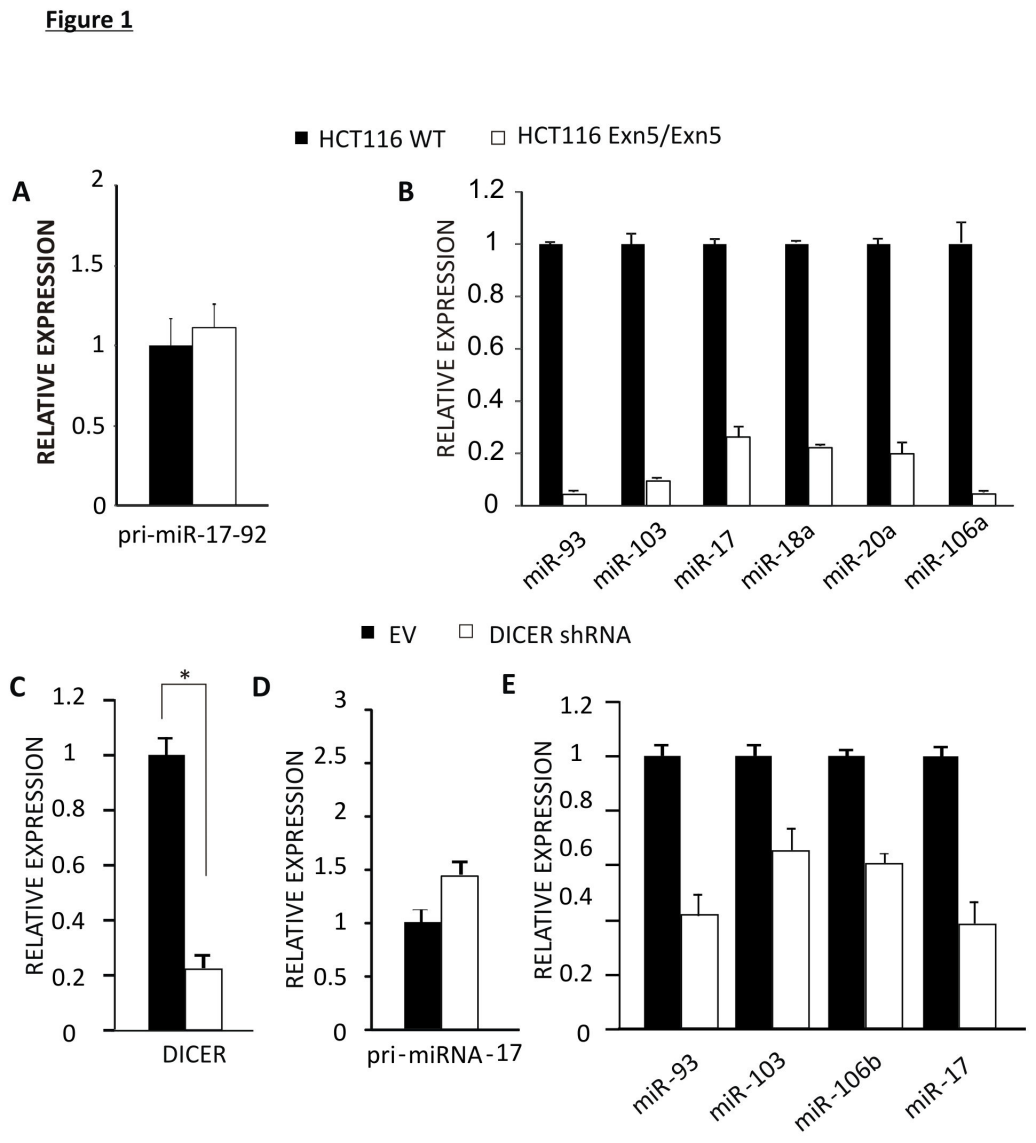
Disruption of DICER impairs miRNA biogenesis. A) QRT-PCR for pri-miR-17 in HCT116 WT and Exn5/Exn5 cells. B) QRT-PCR for miRNAs of the miR-17-92 cluster and others in HCT116 WT and Exn5/Exn5 cells. C) QRT-PCR for Dicer mRNA in HCT116 EV and DICER shRNA cells. D) QRT-PCR for Pri-miRNA-17 in HCT116 EV and DICER shRNA cells. E) QRT-PCR for selected miRNAs in HCT116 EV and DICER shRNA cells.

To investigate the functional significance of this observation, we used HCT116 WT and DICER hypomorphic cells (Exn5 Exn5). Exn5/Exn5 cells have an in-frame 43-amino acid insertion into Exon 5 of the DICER adjacent to DExH motif, an important motif in RNA helicases [40]. The mutant DICER protein has defects in processing of most endogenous pre-miRNAs into mature miRNAs [41]. QRT-PCR showed that although there was no change in pri-miRNA-17 levels as expected (Figure 1 A), there was a significant loss of mature miRNAs belonging to this cluster, such as miR-17, miR-18a, and 20a as well as other miRNAs in Exn5/Exn5 cells when compared to their wild type counterparts (Figure 1 B). Previous characterization of these HCT116 Exn5/ Exn5 cells was carried out by [61] where they have also shown only the mature miRNA expression is compromised in these cells and not their upstream primary and precursor forms. This confirms that the miRNA biogenesis pathway is compromised at the DICER processing step in Exn5/Exn5 cells as expected. To determine whether lower expression of miRNAs was specifically due to compromised miRNA processing and not a nonspecific consequence of the amino acid substitution in the DICER gene, we independently silenced DICER in HCT116 cells using a pSicoR-Dicer shRNA lentivirus. DICER mRNA levels where substantially decreased in pSicoR-DICER shRNA HCT116 cells compared to control vector cells (Figure 1 C). To confirm that any difference in mature miRNA levels observed in DICER shRNA cells can be attributed to specific knockdown of DICER and not are not due to lower expression of primary miRNAs, Pri-miRNA-17 levels were analyzed via QRT-PCR. No decrease in Pri-miRNA-17 was observed in Exn5/Exn5 cells (Figure 1 D). Furthermore, mature miRNA levels were reduced in DICER compromised cells confirming functionality of the knockdown (Figure 1E).

### HCT116 Exn5/Exn5 cells with compromised miRNA biogenesis are resistant to ER stress-induced cell death

**Figure 2:**
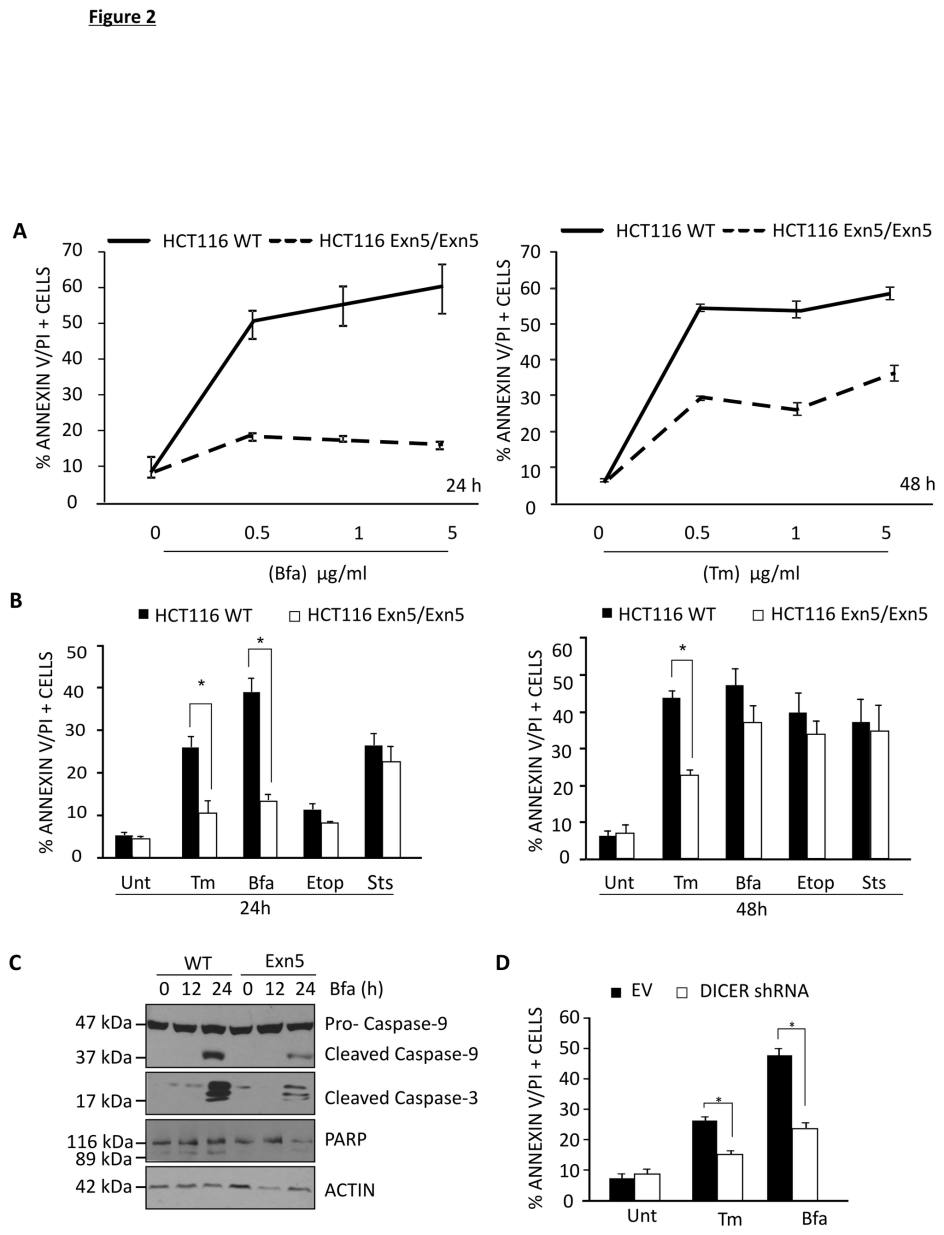
HCT116 cells compromised for miRNA biogenesis are resistant to ER stress-induced cell death. A) HCT116 WT and Exn5/Exn5 cells were treated with a range of concentrations of Bfa for 24 h (LHS) or of Tm for 48 h (RHS). Flow cytometry based measurement of Annexin V/PI positive cells was used to estimate % cell death. B) HCT116 WT and Exn5/Exn5 cells were treated with 500 ng/ml of Tm, 500 ng/ml of Bfa, 100 µM of Etop and 250 nM of Sts for 24 or 48 h. Flow cytometry based measurement, of Annexin V/PI positive cells was used to estimate % cell death. C) Western blots for apoptotic caspases-3 and 9 as well as downstream substrate PARP following treatment of HCT116 WT and Exn5/Exn5 cells with 500 ng/ml of Bfa for 12 h and 24 h. D) HCT116 EV and DICER shRNA cells were treated with 500 ng/ml of Tm and 500 ng/ml of Bfa for 24 h and % cell death was estimated by flow cytometry based measurement of Annexin V/PI.

The consequences of compromising DICER and consequently miRNA biogenesis, were investigated during ER stress. HCT116 Exn5/Exn5 cells exhibited less cell death (Annexin V-FITC and PI positivity) compared to their WT counterparts in response to increasing doses of Bfa (0-5 µg/ml) for 24 h and increasing doses of Tm (0-5 µg/ml) for 48 h. Even at the highest dose of each compound (5 µg/ml) Exn5/Exn5 cells retain sensitivity (Figure 2A). For further experiments 500 ng/ml of Bfa and 500 ng/ml of Tm were used. There was a statistically significant decrease in cell death in Exn5/Exn5 cells treated with 500 ng/ml of Bfa for 24 h (p=0.0005) and 48 h p=0.02) and 500 ng/ml of Tm 24 h (p=0.01) and 48 h p= 0.0001) (Figure 2 B).

In order to determine if the resistance to cell death is ER stress specific phenomenon, cells were also treated with other death inducers, i.e., Etop and Sts. Surprisingly, Exn5/Exn5 cells exhibited only marginal protection to Etop at 24 h (p = ns) and 48 h (p = ns), and Sts for 24 h (p = ns) and 48 h (p = ns) when compared to WT cells (Figure 2 B). Treatment of another colon cancer cell line RKO WT and Exn5/Exn5 cells with 500 ng/ml of Tm (p= 0.05 n=3) and 500 ng/ml of Bfa (p=0.01 n=3) for 24 h also exhibited reduced levels of cell death in the DICER hypomorphic cells, which was not found upon induction of cell death with Etop or Sts (Figure S1). Collectively, these data indicate a specific resistance of Exn5/Exn5 cells to ER stress inducing agents.

Reduced processing of pro-caspase-9, pro-caspase-3 and the caspase substrate PARP was evident in Exn5/Exn5 HCT116 cells following 24 h Bfa treatment confirming protection of these cells against ER stress-induced apoptosis (Figure 2 C). To verify this inhibition was not an artifact of the exon 5 insertion DICER-silenced HCT116 were utilized. Similar to exon5/exon5 HCT116 cells those cells with silenced DICER exhibited significant protection against ER stress-inducing agents Tm (p=0.004 n=3) and Bfa for 24 h (p=0.003) indicating the protection was a general feature of compromised miRNA biogenesis and not specific to the exon 5 insertion method (Figure 2 D).

### HCT116 DROSHA shRNA cells are resistant to ER stress-induced cell death

**Figure 3:**
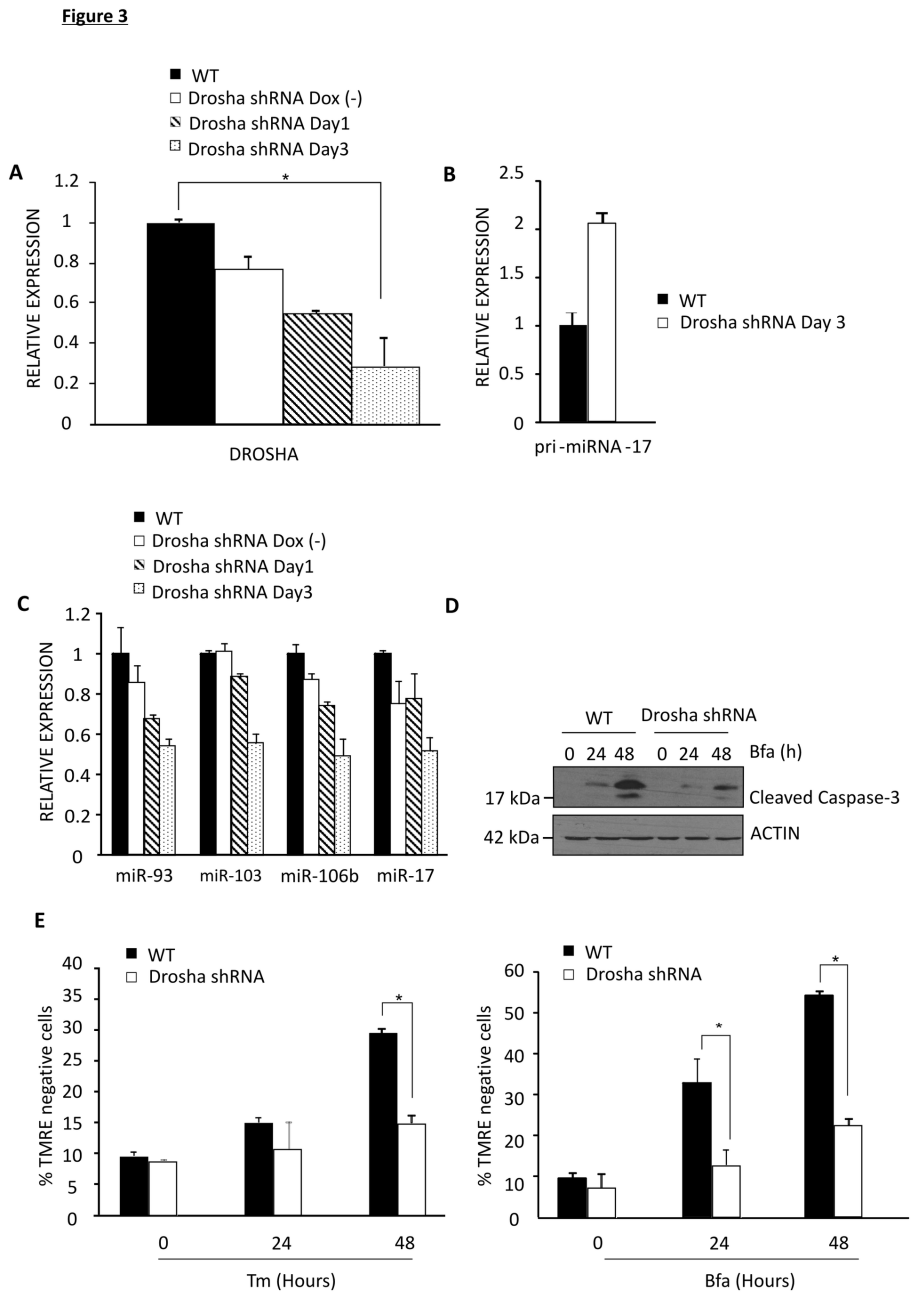
DROSHA compromised HCT116 cells are resistant to ER stress-induced cell death. A) QRT-PCR for Drosha in HCT116 WT, and DROSHA shRNA cells, with or without 500 ng/ml of Doxycycline. B) QRT-PCR for Pri-miRNA-17 WT and DROSHA shRNA cells with Doxycycline for three days. C) QRT-PCR for miRNA in WT and DROSHA shRNA cells with or without Doxycycline. D) Western blot for cleaved caspase-3 following 500 ng/ml of Bfa for 24 h and 48 h in the presence of doxycycline in HCT116 WT and DROSHA shRNA cells. E) HCT116 WT and DROSHA shRNA cells exposed to Doxycycline for 3 days were treated with 500 ng/ml of Tm and 500 ng/ml of Bfa for 24 and 48 h. Flow cytometry based measurement of the percentage of cells that lose TMRE (% TMRE negative cells) was used to indicate the % of cell death.

It has been reported that DICER can have additional functions aside from its role in miRNA biogenesis [42,43]. To ensure the resistant phenotype observed in Exn5/Exn5 and DICER silenced cells is due to compromised miRNA biogenesis, and not a result of interruption of an alternative DICER function, the biogenesis pathway was compromised using a TET-inducible shRNA targeting DROSHA. Doxycycline (500 ng/ml) was added to HCT116 DROSHA shRNA cells for 1 and 3 days after which DROSHA expression was detected via QRT-PCR. This shRNA is leaky and reported to cause some knockdown even in the absence of doxycycline [36]. However, it is clear that there is a further knockdown of DROSHA following three days of doxycycline treatment (Figure 3 A). The levels of pri-miRNA 17 where examined in WT and DROSHA shRNA cells. This was to ensure that any alteration in miRNA biogenesis was not due to events upstream of DROSHA processing. As expected there was no decrease in the expression of pri-miRNA-17 in DROSHA shRNA cells compared to WT (Figure 3 B). Expression of mature miRNA was determined in DROSHA shRNA cells and there was a maximum decrease in miRNA expression following 3 days of Doxycycline (Figure 3 C). Levels of cleaved caspase-3 were reduced in DROSHA shRNA cells compared to WT cells following treatment with ER stress-inducing agents again indicating a block/reduction in ER stress-induced apoptosis (Figure 3 D). Sensitivity of these cells to ER stress-inducing agents Tm and Bfa was determined by examining loss of mitochondrial transmembrane potential (ΔΨM). Similar to Exn5/Exn5 and DICER shRNA HCT116 cells DROSHA shRNA cells displayed statistically significant resistance to ER stress-induced cell death induced by Bfa (24 h p=0.004 n=3, 48 h p=0.005 n=3) and Tm (48 h p=0.02 n=3) as indicated by retention of TMRE positivity (Figure 3 E).

### The unfolded protein response proceeds in the absence of mature miRNA

**Figure 4:**
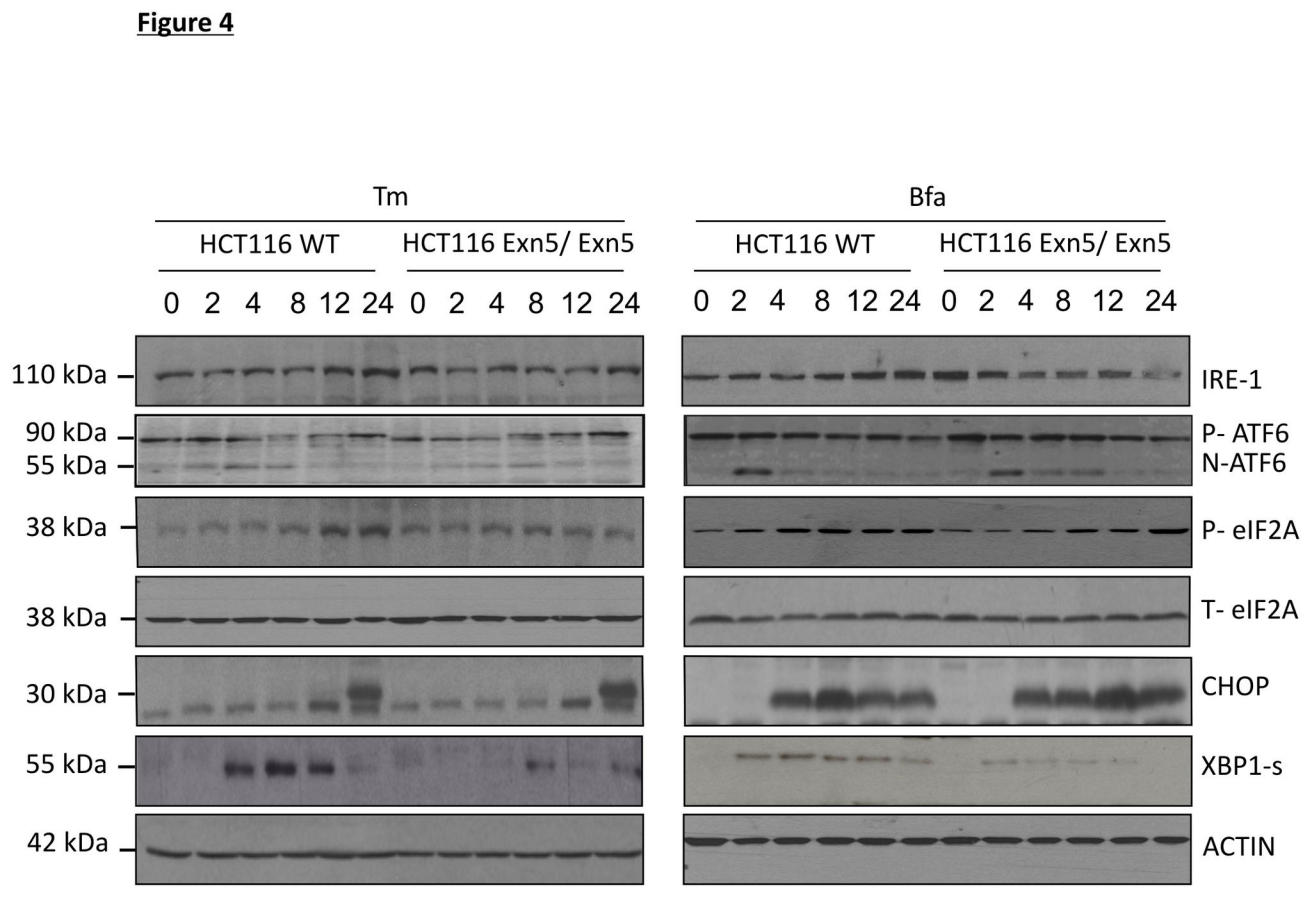
The unfolded protein response is not altered in HCT116 Exn5/Exn5 cells. A) Western blots to detect UPR-associated proteins in HCT116 WT and Exn5/Exn5 cells treated with 500 ng/ml of Bfa and 500 ng/ml of Tm for 2, 4, 8, 12 and 24 h. Proteins analyzed include IRE1α, ATF6, P-eIF2α and T-eIF2α, CHOP, XBP1s. ACTIN was used a loading control. The data is a representative of at least three independent experiments.

As Exn5/Exn5 cells were resistant to ER stress, we examined levels of UPR proteins to determine if differences existed between WT and Exn5/Exn5 cells. Levels of IRE1, ATF6, phospho-eIF2α (P-eIF2α), total- eIF2α (T-eIF2α), CHOP, and XBP1s were examined by Western blotting. No difference in UPR protein expression between WT and Exn5/Exn5 cells was observed, with the notable exception of spliced XBP-1 (XBP1s) whose levels were consistently decreased in Exn5/Exn5 cells (Figure 4). This was surprising since prolonged XBP1s levels are associated with cell survival. To determine if reduced XBP1s contributed to the resistant phenotype of Exn5/Exn5 cells an inhibitor of IRE1, MKC4485, was utilized. MKC4485 binds to and specifically blocks the RNase domain of IRE1 thus abrogating XBP1 splicing [44]. Titration of MKC4485 indicated 5 µM was sufficient to prevent ER stress-induced splicing of XBP1 (Figure S2 A). Using MKC4485 we examined the effect of blocking XBP-1s on the cellular response to ER stress-inducing agents. Addition of MKC4485 to HCT116 cells treated with ER stress-inducing agents failed to mimic the protection evident in DICER compromised HCT116 cells (Figure S2 B). The inability of MKC4485 to reduce cell death in HCT116 cells indicates IRE1 RNase activity does not contribute to the observed protection despite DICER compromised cells exhibiting lower levels of XBP1s upon induction of ER stress (Figure 4). Other aspects of IRE1 signaling, such as its kinase activity, may contribute to ER stress-induced death. IRE1 is known to activate JNK signaling, an effect that should not be altered upon addition of MKC4485 which only targets the RNase domain of IRE1 while JNK is activated through the IRE1-kinase signaling. Hence, WT and Exn5/Exn5 cells treated with 500 ng/ml of Bfa for 2-24 h were examined for JNK phosphorylation which is thought to be activated through IRE1 kinase signaling and TRAF2. No differences in total or phospho-JNK between WT and Exn5/Exn5 cells were observed upon treatment with ER stress-inducing agents indicating IRE1 signaling does not contribute to the resistance of Exn5/Exn5 cells (Supplementary Figure S2 C).

### Resistance of Exn5/ Exn5 cells occurs upstream of the mitochondria

**Figure 5:**
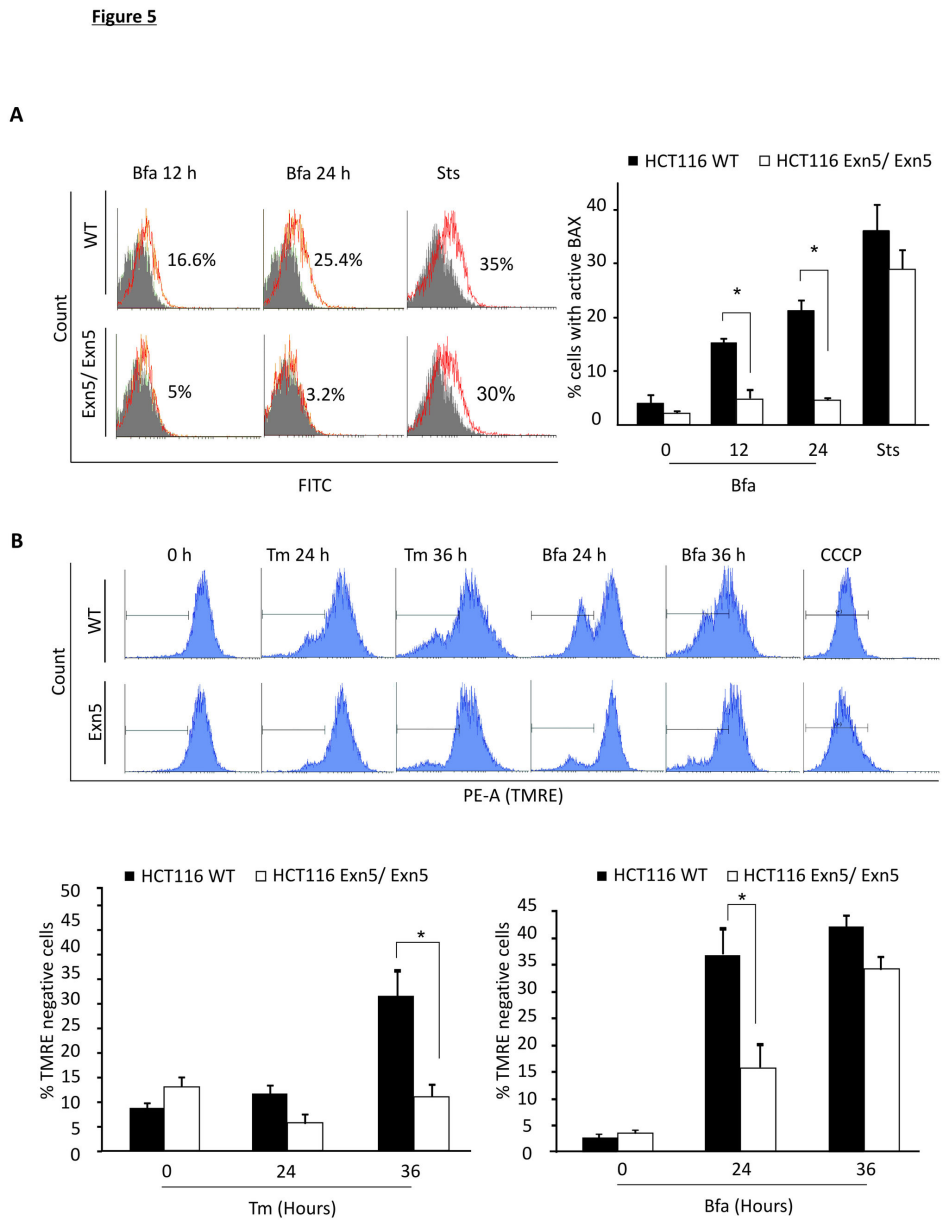
Resistance of Exn5/Exn5 cells occurs upstream of the mitochondria. A) Flow cytometry based measurement of the active conformation of BAX in HCT116 WT and Exn5/Exn5 cells treated with 500 ng/ml of Bfa for 12 and 24 h or 100 nM Sts for 24 h. Histogram overlays were used to represent the data shown, where treated samples (red peak) were compared to untreated samples (Grey peak). Exn5/Exn5 cells do not become FITC positive indicating that these cells have less active BAX then WT cells. The graphed representation of % cells with active BAX is also shown (RHS). B) Flow cytometry profiles of TMRE staining in HCT116 WT and Exn5/Exn5 cells treated with 500 ng/ml of Bfa and 500 ng/ml of Tm for 24 and 36 h. Histogram plots (blue) show loss of TMRE staining in the PE channel. The graphed representation of the percentage of cells that lose TMRE (% TMRE negative cells) is shown to represent the percentage of dying cells (lower graphs).

Our results suggest that while miRNAs do not play a role in the activation of the UPR response they may have a role further downstream in the execution of ER stress-induced cell death pathways. ER stress-induced cell death has previously been reported to signal through the mitochondrial or the intrinsic cell death pathway [2]. To determine the role of mitochondria-mediated death processes in our system we examined the translocation of BAX from the cytoplasm to the mitochondria using a conformation specific antibody that specifically detects the active form of BAX [45]. Reduced levels of active BAX were detected in Exn5/Exn5 cells treated with 500 ng/ml Bfa for 12 h (p=0.05 n=3) and 24 h (p= 0.026 n=3) (Figure 5 A) suggesting a possible impairment in mitochondrial mediated death signals. This idea was further supported by examining ΔΨm. A significant retention of ΔΨm was evident in Exn5/Exn5 cells when compared to WT cells, following 36 h Tm treatment (p=0.042 n=3) and 24 h treatment with Bfa (p= 0.012 n=3) again indicating an impairment in mitochondria mediated death signaling (Figure 5 B).

### Altered expression of BCL-2 family of protein may attenuate cell death in Exn5/Exn5 cells

**Figure 6:**
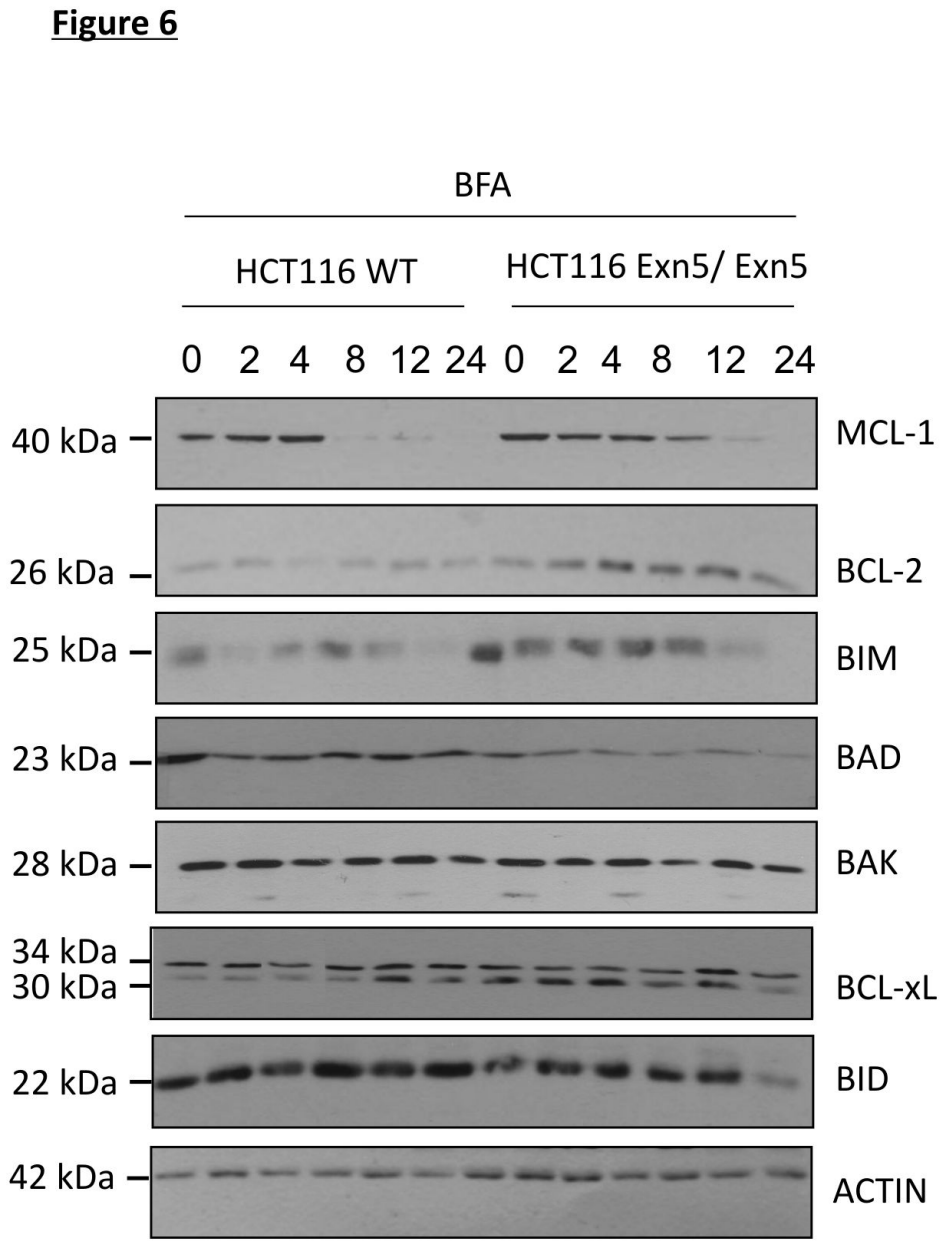
Altered expression of the BCL-2 family of proteins may attenuate cell death in Exn5/Exn5 cells. A) Western blots for BCL-2 family proteins in HCT116 WT and Exn5/Exn5 cells treated with 500 ng/ml of Bfa for 2, 4, 8, 12 and 24 h. Proteins analyzed were MCL-1, BCL-2, BIM, BAD, BAK, BCL-xL, BID and NOXA. ACTIN was used as a loading control. The data is a representative of at least three independent experiments.

The balance between pro-survival and pro-apoptotic BCL-2 family proteins, is known to modulate mitochondrial apoptosis. Since there is less BAX activation in Exn5/Exn5 cells, we compared the levels of BCL-2 family members in WT and Exn5/Exn5 cells during 0-24 h treatment with 500 ng/ml of Bfa. The expression levels of pro-survival BCL-2 family members, MCL-1 and BCL-2, were prolonged and higher, respectively in Exn5/Exn5 cells while levels of pro-apoptotic BAD were much lower in these cells. Unexpectedly, Exn5/Exn5 cells displayed elevated BIM levels yet exhibit significant protection against ER stress-induced cell death was observed. No notable difference in expression of BAK, BCL-xL, BID or NOXA was observed between the two cell lines (Figure 6). These data suggests that the balance of pro and anti-apoptotic BCL-2 family proteins may differ in favor of pro-survival members in Exn5/Exn5 cells compared to WT cells and this may account for the observed inhibition in ER stress-induced death.

## Discussion

This study describes a previously unknown functional role for miRNA biogenesis machinery in ER stress-induced apoptosis. Using HCT116 and RKO Exn5/Exn5 cells, and DICER silenced HCT116 cells we consistently observed resistance against ER stress inducing stimuli. This impairment in cell death was also evident upon silencing of DROSHA, verifying it was not specifically restricted to DICER disruption but rather is a general feature of compromised miRNA biogenesis. Furthermore, a global loss of miRNAs interferes with ER stress-induced modulation of the BCL-2 family, resulting in sustained expression levels of anti-apoptotic proteins such as BCL-2 and MCL-1. As a consequence of this, mitochondria remain intact, preventing the release of pro-apoptotic factors thereby reducing cell death.

These findings have relevance to cancer where miRNAs or DICER and other components of the miRNA biogenesis machinery are increasingly implicated in disease pathogenesis, due to altered expression [46,47]. Loss of DICER is associated with increased tumorigenicity and with poor patient prognosis in several cancers [25–27], DICER downregulation has been reported in colorectal cancer with low DICER expression associated with advanced, high grade tumors and poor patient outcome [48]. How loss of miRNAs promotes tumor development is not fully understood. Our results indicate downregulation of miRNAs specifically inhibits/attenuates stress-induced cell death. This may be particularly important in tumor development where cells are frequently subjected to ER stress within the tumor microenvironment. In such instances downregulation of miRNAs may provide tumor cells with a means by which to escape cell death initiation. Following the initiation of malignancy, there is poor vascularization of the tumor mass which leads to stressful conditions in the tumor microenvironment, including hypoxia, nutrient deprivation and pH changes. These conditions activate a range of cellular stress-response pathways, including the UPR [49]. Perhaps tissues with low DICER expression are better adapted to cope with stressful conditions and thus may have an increased capacity for survival.

Exn5/Exn5 cells did not display major alterations in the expression pattern of UPR marker proteins but their main apoptotic pathway is altered. In theory, the same cells should be resistant to other inducers of apoptosis but protection was only conferred to Tm and Bfa treatments. It is well known that cells can respond very differently to various stress stimuli. Therefore we hypothesize that Etop or Sts regulate a different subset of miRNAs and as such proteins that enhance survival of cells in response to ER stress may be differentially regulated by Etop or Sts-induced cell death, and thus would not provide protection to these treatments. In addition to this DICER can promote or deter survival depending on cell type or stimulus, suggesting that miRNAs are vital for the death promoting signals in a stimulus-dependent maner. Also compounds such as Etop and Sts are known to activate many signaling pathways to induce intrinsic cell death [50–53]; while Tm and Bfa only signal through the UPR. Loss of miRNA may not confer resistance to Tm and Bfa due to some level of redundancy. For instance, Etop can activate p53 and PKCδ to activate caspase-3 [54], but cells lacking p53 and BAX can still undergo Etop-induced apoptosis through p73 and BID [55]. It is likely that the BCL-2 proteins are modulated differently in response to different stimuli. In fact, Etop can decrease BCL-xL [56] or dephosphorylate BCL-2 to promote cell death [57]. This may explain why Exn5/Exn5 cells are more specifically resistant to ER stress-induced cell death and not death induced by Etop or Sts.

Many reports describing the regulation of miRNA during apoptosis, indirectly show that such regulation occurs upstream of the mitochondria at the level of the BCL-2 family of proteins [34]. Our study shows that loss of DICER and thus mature miRNAs modulates the expression of BCL-2 family members with higher anti-apoptotic BCL-2 and MCL-1 in DICER compromised cells. Global loss of mature miRNA in this modal would attenuate availability of miRNAs that repress pro-survival molecules during apoptosis. For example miR-17-92 or miR-15a-16-1 normally target BIM and BCL-2 respectively, so loss of these miRNAs may explain the elevated levels of these proteins in response to ER stress. BCL-2 family proteins are thought to act as a rheostat with pro- and anti-apoptotic members competing with each other to either promote or block apoptosis [7,58]. Our results suggest that loss of DICER causes higher or sustained levels of pro-survival BCL-2 and MCL-1 that may neutralize pro-apoptotic BH3-only proteins in response to ER stress, delay mitochondrial apoptosis and thus, enhance survival. Surprisingly we also observed consistently higher levels of BIM in Exn5/Exn5 cells, which was unexpected for cells that exhibit protection to ER stress. BIM is conventionally a pro-apoptotic protein and is an important inducer of ER stress induced cell death [59]. Therefore, a high level of BIM in a model resistant to ER stress does raise questions as to why these cells are not dying. The rheostat model proposed above can further be supported by the study reported higher BIM expression in prostate and breast cancer but BIM was phosphorylated at Ser69 and Ser87, which are thought to neutralize the apoptotic function of BIM, furthermore, BIM was sequestered by pro-survival BCL-xL and MCL-1 [60].

A correlative study of human cancer samples with DICER expression, tumor progression and levels of BCL-2 family of proteins would help shed further light on our findings. We hypothesize that cancers with low DICER, and enhanced tumorigenesis could have altered BCL-2 family expression to enhance survival.

## Supporting Information

Figure S1 **RKO WT and Exn/Exn5 cells are resistant to ER stress-induced cell death.**
A) QRT-PCR for selected miRNAs in RKO WT and Exn5/Exn5 cells. B) RKO WT and Exn5/Exn5 cells treated with 500 ng/ml Tm, 500 ng/ml Bfa, 100 µM Etop and 250 nM Sts for 24 h. Flow cytometry based measurement of Annexin V/PI positive cells was used to estimate % cell death.
(TIF)

Figure S2 **IRE1 signaling is not important for the resistant phenotype of HCT116 Exn5/ Exn5 cells.**
A) HCT116 WT cells with or without increasing doses of IRE1 inhibitor MKC4485 were treated with 500 ng/ml of Bfa for 24 h to determine the minimum effective dose at which MKC4485 inhibits splicing of XBP1. B) HCT116 WT and Exn5/Exn5 cells were treated with and without 5 µM of MKC4485 and 500 ng/ml of Bfa or 500 ng/ml of Tm for 24 h and MKC4485 alone. Flow cytometry based measurement of Annexin V/PI positive cells was used to estimate % cell death. C) Western blots for T-JNK and P-JNK proteins in HCT116 WT and Exn5/Exn5 cells treated with 500 ng/ml of Bfa for 2, 4, 8, 12 and 24 h. ACTIN was used as a loading control.
(TIF)
