# Supplementary figures and images for: Disruption of microRNA Biogenesis Confers Resistance to ER Stress-Induced Cell Death Upstream of the Mitochondrion
Source: PLoS One. 2013 Aug 19;8(8):e73870. doi: 10.1371/journal.pone.0073870 (PMC3747093; doi:10.1371/journal.pone.0073870)

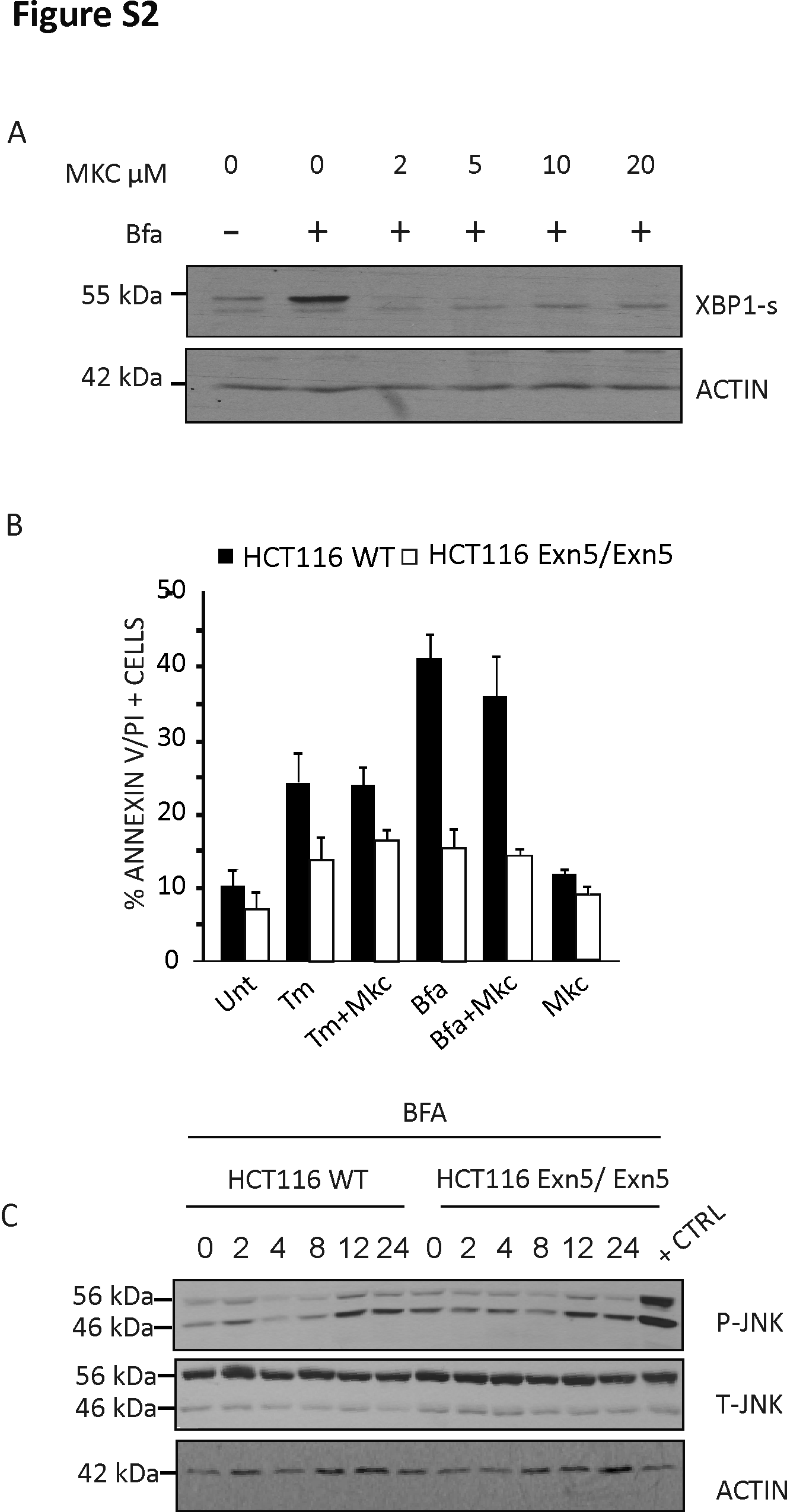

Supplement: Figure S2 — IRE1 signaling is not important for the resistant phenotype of HCT116 Exn5/ Exn5 cells. A) HCT116 WT cells with or without increasing doses of IRE1 inhibitor MKC4485 were treated with 500 ng/ml of Bfa for 24 h to determine the minimum effective dose at which MKC4485 inhibits splicing of XBP1. B) HCT116 WT and Exn5/Exn5 cells were treated with and without 5 µM of MKC4485 and 500 ng/ml of Bfa or 500 ng/ml of Tm for 24 h and MKC4485 alone. Flow cytometry based measurement of Annexin V/PI positive cells was used to estimate % cell death. C) Western blots for T-JNK and P-JNK proteins in HCT116 WT and Exn5/Exn5 cells treated with 500 ng/ml of Bfa for 2, 4, 8, 12 and 24 h. ACTIN was used as a loading control. (TIF) [file pone.0073870.s002.tif]
